# Supplementary material for: MetaQTL: a package of new computational methods for the meta-analysis of QTL mapping experiments
Source: BMC Bioinformatics. 2007 Feb 8;8:49. doi: 10.1186/1471-2105-8-49 (PMC1808479; doi:10.1186/1471-2105-8-49)
Supplement: Additional File 7 — MetaQTL Package : jar file and tutorial. This Zip archive contains both the MetaQTL JAR file and the files of the tutorial. [file 1471-2105-8-49-S7.zip › org.inra.metaqtl/doc/org/metaqtl/class-use/Qtl.html]

Uses of Class org.metaqtl.Qtl


|  |  |  |
| --- | --- | --- |
| **Overview** \| **Package** \| **Class** \| **Use** \| **Tree** \| **Deprecated** \| **Index** \| **Help** | |  |
| PREV   NEXT | **FRAMES**    **NO FRAMES**     **All Classes** |  |


---


## **Uses of Class org.metaqtl.Qtl**

| Packages that use Qtl | |
| --- | --- |
| **org.metaqtl** |  |
| **org.metaqtl.adapter** |  |

Uses of Qtl in org.metaqtl

| Fields in org.metaqtl declared as Qtl | |
| --- | --- |
| `Qtl[]` | `Chromosome.mqtls`             The meta Qtls on this chromosome. |
| `Qtl[][]` | `MetaQtlAnalysis.qtlByChrom`             The matrix of qtl by chromosome |
| `Qtl[]` | `Chromosome.qtls`             The QTLs located on this chromosome. |
| `protected  Qtl[]` | `MetaQtlData.qtls`             The QTL. |

| Methods in org.metaqtl that return Qtl | |
| --- | --- |
| `Qtl` | `Qtl.getCopy()`             Returns a copy of the QTL. |
| `Qtl[]` | `MetaQtlResult.getMetaQtl(int k)` |
| `Qtl[]` | `MetaQtlAnalysis.getMetaQtl(java.lang.String chrom, java.lang.String trait, int k)`             Returns the meta Qtl for the given chromosome indice `cidx`, the given trait name `trait` and the given number of clusters `k`. |
| `Qtl[]` | `Chromosome.getMetaQtls()` |
| `Qtl` | `MetaQtlData.getQtl(int i, int j)`             Returns the j^th QTL in the i^th cluster. |
| `Qtl[]` | `Chromosome.getQtls()`             This method returns a copy of the QTL located on thic chromosome. |

| Methods in org.metaqtl with parameters of type Qtl | |
| --- | --- |
| `void` | `Chromosome.attachQtl(Qtl[] qtls)` |
| `double` | `Chromosome.getQTLInMrkIntProb(int mrkIdxL, int mrkIdxR, Qtl qtl)`             This methods returns the probability that the QTL be inside the marker interval with the given right and left marker indices and the given QTL. |
| `void` | `Chromosome.setMetaQtls(Qtl[] metaQtl)` |

Constructors in org.metaqtl with parameters of type Qtl

`MetaQtlData(Qtl[] qtls)`
  
          Create a new instance of `MetaQtlData` which
will represent the given array of `Qtl`.

Uses of Qtl in org.metaqtl.adapter

| Methods in org.metaqtl.adapter that return Qtl | |
| --- | --- |
| `static Qtl[]` | `QtlAdapter.adapt(EMResult result)`             From a result of a EM clustering returns the mixture components as an array of Qtl. |
| `static Qtl` | `QtlAdapter.toQTL(IBioLocus locus)`             This method converts an object which class implements the `IBioLocus interface into a Qtl.` |

| Methods in org.metaqtl.adapter with parameters of type Qtl | |
| --- | --- |
| `static QtlPartition` | `QtlPartitionAdapter.adapt(MetaQtlResult result, Qtl[] qtls, int k)` |
| `static IBioLocus` | `QtlAdapter.fromQTL(Qtl qtl)`             This method converts a `Qtl` into an object which class implements the `IBioLocus interface.` |

---


|  |  |  |
| --- | --- | --- |
| **Overview** \| **Package** \| **Class** \| **Use** \| **Tree** \| **Deprecated** \| **Index** \| **Help** | |  |
| PREV   NEXT | **FRAMES**    **NO FRAMES**     **All Classes** |  |


---
